# Bacillus Calmette-Guerin (BCG): the adroit vaccine

**DOI:** 10.3934/microbiol.2021007

**Published:** 2021-02-08

**Authors:** Oluwafolajimi A. Adesanya, Christabel I. Uche-Orji, Yeshua A. Adedeji, John I. Joshua, Adeniyi A. Adesola, Chibuike J. Chukwudike

**Affiliations:** 1 Institute for Advanced Medical Research and Training (IAMRAT), College of Medicine, University of Ibadan, Ibadan, Nigeria; 2 Department of Medicine, College of Medicine, University of Ibadan, Ibadan, Nigeria

**Keywords:** BCG, non-specific effects, vaccine, tuberculosis, COVID-19

## Abstract

**Background:**

The Bacillus Calmette-Guerin (BCG) vaccine has been in use for 99 years, and is regarded as one of the oldest human vaccines known today. It is recommended primarily due to its effect in preventing the most severe forms of tuberculosis, including disseminated tuberculosis and meningeal tuberculosis in children; however, its efficacy in preventing pulmonary tuberculosis and TB reactivation in adults has been questioned. Several studies however have found that asides from its role in tuberculosis prevention, the BCG vaccine also has protective effects against a host of other viral infections in humans, an effect which has been termed: heterologous, non-specific or off-target.

**Objectives:**

As we approach 100 years since the discovery of the BCG vaccine, we review the evidence of the non-specific protection offered by the vaccine against viral infections, discuss the possible mechanisms of action of these effects, highlight the implications these effects could have on vaccinology and summarize the recent epidemiological correlation between the vaccine and the on-going COVID-19 pandemic.

**Results:**

Several epidemiological studies have established that BCG does reduce all-cause mortality in infants, and also the time of vaccination influences this effect significantly. This effect has been attributed to the protective effect of the vaccine in preventing unrelated viral infections during the neonatal period. Some of such viral infections that have been investigated include: herpes simplex virus (HSV), human Papilloma virus (HPV), yellow fever virus (YFV), respiratory syncytial virus (RSV) and influenza virus type A (H1N1). These effects are thought to be mediated via induction of innate immune memory as well as heterologous lymphocytic activation. While epidemiological studies have suggested a correlation, the potential protection of the BCG vaccine against COVID-19 transmission and mortality rates is currently unclear. Ongoing clinical trials and further research may shed more light on the subject in the future.

**Conclusion:**

BCG is a multifaceted vaccine, with many numerous potential applications to vaccination strategies being employed for current and future viral infections. There however is a need for further studies into the immunologic mechanisms behind these non-specific effects, for these potentials to become reality, as we usher in the beginning of the second century since the vaccine's discovery.

## 1. Introduction

Following over 13 years of research by Albert Calmette and Camille Guerin of the French Pasteur Institute, the Bacillus Calmette-Guerin (BCG) vaccine was finally introduced in 1921 as an attenuated live vaccine of *Mycobacterium bovis* [1]. The vaccine described by Calmette and Guerin was obtained following serial sub-culturing (231 passages) on glycerinated bile potato medium, and the first dose of the vaccine was administered orally, and this continued untill the 1970s when this was replaced by the intradermal route, now recommended by the World Health Organization (WHO) [2],[3]. With over 4 billion administered doses worldwide, and an additional 100 million newborns being vaccinated annually, BCG is currently the most widely used vaccines in the world; however, there exists no universal or global BCG vaccination policy, as various countries have taken differing approaches to vaccinating their populations [4]. While countries like the United Kingdom as well as most countries in Africa and Asia have employed universal BCG vaccination schemes, others such as the United States of America (USA), Canada, Italy, Belgium and the Netherlands, have either recommended BCG vaccination for high-risk demographics, or have never had any form of BCG vaccination policy in place [5].

The BCG vaccine offers protection against disseminated TB and meningeal TB in children [6],[7], however, its efficacy against pulmonary TB in adults ranges from 0% to 80%, and is dependent on several factors including: geographical location, vaccine strain used and previous exposure to environmental mycobacterial pathogens [8]. There are existing evidences that this vaccine originally developed to protect against TB, also offers protection against non-tuberculous mycobacterial (NTM) infections such as the Buruli ulcer disease, and more importantly, leprosy [9], and in an interesting turn of events, following its introduction, several epidemiological studies [10]–[14] published began to suggest that the BCG vaccine had a significant effect in reducing infant mortality, in a way that was in fact too large to be explained by its anti-mycobacterial action alone. These eventually paved way for many more *in vitro, in vivo* as well as clinical studies which have shown that the BCG vaccine does have certain ‘heterologous’ or ‘non-specific’ or ‘off-target’ effects against other pathogenic organisms, especially viruses, which are beneficial and have been linked to the vaccine's effect of causing a reduction in all-cause mortality.

The aim of this article is to present a review of the many ‘faces’ of the BCG vaccine, by reviewing the evidences in literature of the effect of BCG vaccination on all-cause mortality, as well as on viral infections in both animal model experiments and in human trials. We then speak about the several mechanisms that have been suggested to explain these non-specific effects, involving both the innate and adaptive immune systems; we highlight the implications of these heterologous effects for the field of vaccinology; and we conclude by evaluating the proposed correlation certain epidemiological studies have drawn between BCG vaccination and SARS-CoV-2 transmission as well as morbidity and mortality figures. We conducted our literature search using the PubMed, Google Scholar and HINARI databases, and we employed a combination of terms including: BCG, non-specific effects, heterologous effects, trained immunity, vaccine and tuberculosis.

## 2. Effect of BCG vaccination on all-cause mortality

Soon after the first dose of the BCG vaccine was administered in 1921, several countries began to roll out universal BCG vaccination schemes for their population. These allowed for many epidemiological studies to be conducted with the aim of evaluating the efficacy of the vaccine in reducing TB mortality in the vaccinated populations. Interestingly however, these studies soon discovered that the vaccine was able to reduce infant mortality to a degree larger than could be explained by its effect on TB alone [10],[11]. In several West African studies carried out in Guinea-Bissau, the authors observed a 50% reduction in all-cause morbidity and mortality in BCG vaccinated children, as against unvaccinated children [12]–[14]. In an observational follow-up study by Kristensen *et al*. [12] they found that BCG vaccinated children (between 6 and 20 months old) had a significantly lower mortality ratio of 0.55 when compared with the unvaccinated cohort which had a mortality ratio of 0.74. These findings were corroborated by Roth *et al*. in their cohort study, which monitored the mortality pattern for children (3 to 6 months old) with and without a BCG scar [13]. The study was carried out in Bandim I, Bandim II, Belem, and Mindara which are all districts of Bissau, the capital of Guinea Bissau. Participants were divided into two cohorts; A and B. In the former, consisted of children recruited at six months of age, with the BCG scar and had been vaccinated at least a month prior, while the latter, consisted of children between three months to five years with the BCG scar and had been vaccinated at least a month before scar reading [13]. These studies suggested that the fall in all-cause mortality could be attributed to a reduction in the occurrence of neonatal sepsis and subsequent respiratory infections in the BCG vaccinated children. Similar observations have been reported in randomized controlled trial from Guinea-Bissau [15], and a prospective cohort study from Uganda [16], as well as a WHO Special Advisory Group systematic review by Zimmermann *et al*. [17], which showed a 30% reduction in all-cause mortality (RR 0.70; CI 95%, 0.49–1.01), following neonatal BCG vaccination.

Two randomized controlled trials from Guinea-Bissau, comparing the mortality rates in cohorts of children who were either vaccinated at birth or had delayed vaccination, also presented interesting results. Aaby *et al*. [18] reported that children who were vaccinated within 3 days had a lower mortality rate ratio of 0.49 (0.21–1.15) compared with a ratio of 0.55 (0.34–0.89) in those who were vaccinated after 4 weeks. While Biering- Sørensen *et al*. [19] reported a mortality rate ratio of 0.17 (0.02–1.35) in children vaccinated within 3 days after birth and 0.28 (0.06–1.37) in children vaccinated after 3 days but within the first 4 weeks. These two trials provided evidence to suggest that the time of BCG vaccination may also influence the resulting effect of the vaccination on mortality rate. This however contradicts findings from another randomized controlled trial by Stensballe *et al*. [20] in a sample of the Danish population, which suggested that BCG vaccination had no effect on reducing all cause hospitalization, untill about 15 months of age. In their study, Stensballe *et al*. [20] noted that the mean rate of hospitalization (up to 15 months of age) in both the BCG vaccinated and non-BCG vaccination groups were 0.49 and 0.47 hospitalizations per child respectively. This apparent difference in results between the developing and developed population groups may be attributed to various environmental factors such as poor hygiene in the developing countries, which make children more susceptible to infections and thus allows BCG vaccination to present a more profound and earlier effect in reducing all-cause hospitalizations and subsequent mortality.

The summary of these clinical evidence prove that BCG does indeed have beneficial effects independent from its effect against tuberculosis. These effects are termed ‘heterologous’ or ‘non-specific’ or ‘off-target’ effects, and they have been hypothesized to offer protection against non-mycobacterial pathogens such as: other bacteria and even viruses.

## 3. Effect of BCG vaccination on viral infections in Humans

### 3.1. For prophylaxis

The clinical trials and evidences discussed up untill now have only involved all-cause mortality and not differentiated between bacterial or viral causes of infection. However, it has been suggested that the non-specific effects of the vaccine would be mediated mainly through the prevention of viral infections which are quite common during the neonatal period. To provide evidence of this, Stensballe *et al*. [21] carried out a case-control study investigating the impact of BCG vaccination on the prevention of acute lower respiratory tract infection caused by the respiratory syncytial virus (RSV) in infants in Guinea-Bissau. Their results provided evidence to suggest that BCG vaccination was associated with lower odds of RSV infection, compared with unvaccinated infants, with the effect being more marked in girls [21]. Conversely, Wardhana *et al*. [22] embarked on a randomized controlled trial to investigate the efficacy of BCG vaccination to prevent acute lower respiratory tract infections in the elderly in Indonesia. They administered the BCG vaccine once a month for 3 months to a cohort of 60–75 year olds in their test group, while the control group received placebo. At the end of the study period, they noted a significant reduction in the prevalence of acute upper respiratory tract infection in the BCG group, as well as a significantly higher level of IFN-γ [22]. Furthermore, a Japanese clinical trial, the authors note that BCG vaccination of tuberculin-negative elderly patients resulted in a decrease in the risk of pneumonia [23].

### 3.2. For Immunotherapy

Besides offering prophylactic protection against non-mycobacterial infections, the heterologous effects of the BCG vaccine have also been shown to have immunotherapeutic potential against clinical conditions mediated by viral infections. In 2013, Salem *et al*. [24] reported the findings of a randomized placebo controlled clinical trial they performed, to investigate the safety and efficacy of topical BCG for the treatment of common and plane warts caused by the Human Papilloma Virus (HPV) in children. They noted a significant difference (*p* < 0.001) in outcomes in both the test and control groups, with as much as 65% of children with common warts and 45% with plane warts in the test groups recovering completely from the condition, with no recurrences or side effects recorded during the 6 months follow up period [24]. No participant in the placebo control group recovered during the study period. Another randomized double-blind, controlled study in India, by Podder *et al*. [25], investigating the efficacy of BCG against viral warts, also noted a statistically significant difference in results between BCG vaccinated and unvaccinated groups, with complete clearance being recorded in 48.5% (16 out of 33) of patients in the BCG vaccinated cohort (*p* < 0.001). In a series of seven cases of recurrent viral warts treated with BCG immunotherapy, by Daulatabad *et al*. [26], they showed that a single dose of BCG caused regression of warts in 85.7% of patients and complete clearance in 28.6% of patients. They however noted the development of adverse effects such as: pain over injection site, scars and abscesses, in 57.1% of their patients upon the administration of repeated doses of the vaccine, raising concerns about its safety profile.

In addition, intravesical BCG immunotherapy as an adjuvant treatment for non-muscle invasive bladder cancer was first described by Morales *et al*. [27] in 1972, with over 3 million treatment courses being administered annually. The mechanism of the anti-tumor effect of BCG, though not completely understood, was described by Jackson and James in 1994 [28], to consist of a tumor response and an eventual immune response. According to them, the tumor response is characterized by changes in the phonotypic characteristics of the tumor cells, secondary to cytokine stimulation, especially by IFN-γ following BCG administration [28]. The two most important of these phenotypic changes include the expression of MHC class II molecules on the tumor cells, as first described by Prescott *et al*. [29] in 1989, and the expression of intracellular adhesion molecule-1 (ICAM-1) on the tumor cells, following BCG administration [30]. The immune response is characterized by the infiltration by both CD4 and CD8 T-cells, with CD4 T-cells, following activation by MHC class II on the surface of the tumor cells, secreting cytokines that cause the maturation of the CD8 T-cells [28]. These CD8 T-cells then kill the tumor cells by triggering apoptosis, following activation by MHC class I and intracellular adhesion molecules (ICAM) on tumor cells. The immune cells then detach from the destroyed tumor cells and are ready to repeat the process again. It is important to note however, that due to the role of the immune cells, especially T-cells in mediating the immunotherapeutic effects of BCG against superficial bladder cancer, an intact immune system is required for this therapy to be effective [31].

### 3.3. As a vaccine adjuvant

Furthermore, the BCG vaccine has also been shown to improve the immune response to other unrelated vaccines against viral infections, administered later in life. In a randomized controlled trial involving 20 test subjects who received the BCG vaccine and 20 control subjects who received placebo, with all subjects receiving the trivalent influenza vaccine 14 days later, the hemagglutination-inhibiting (HI) antibodies response to the 2009 pandemic influenza A (H1N1) vaccine in the BCG vaccinated group was significantly higher in the test group, than in the placebo group [32], suggesting that the BCG vaccine does enhance the immunogenicity of subsequently administered influenza vaccines. A similar adjuvant-like effect has been shown for other vaccines against viral infections like the hepatitis B virus (HBV) [33]–[35] and wild polio virus (WPV) [34]. In their trial, Ritz *et al*. [33], found that the same result of an enhanced immune response was obtained from the use of other vaccines against bacterial infections such as: *Haemophilus influenza* type B, *Clostridium tetani* (tetanus toxoid) and *Streptococcus pneumoniae* (pneumococcal conjugate vaccine –PCV). Since the extent of immunologic response mounted following vaccination can be taken to be directly proportional to the level of protection offered by a vaccine, the ability of BCG to serve as an adjuvant for other unrelated vaccines may be taken as an indirect evidence of its ability to protect against these infections. Table 1 gives an overview of the various non-specific effects BCG has on viral infections in humans, with the corresponding evidence in literature.

**Table 1.** Overview of the non-specific effects of the BCG vaccine on human viral infections.

Virus	Study type	Effect of BCG recorded	Reference	Sample size
Yellow fever virus (YFV)	Randomized controlled trial (RCT)	Reduction in yellow fever vaccine viraemia, which was positively associated with IL-1ß production	Arts *et al*. [36]	-
Human Papilloma virus (HPV)	Randomized controlled trial (RCT)	Improved clearance of the HPV caused viral warts	Salem *et al*. [24]	80 participants (randomized into 40 test and 40 control subjects)
	Randomized controlled trial (RCT)		Podder *et al*. [25]	60 participants (randomized into 33 participants in BCG group and 27 in tuberculin purified protein group
	Case series		Daulatabad *et al*. [26]	7 cases (wart regression seen in 85.7% of patients and complete resolution in 28.6%)
Respiratory syncytial virus (RCV)	Case-control study	Fewer cases of RSV infection in BCG vaccinated children in Guinea-Bissau	Stensballe *et al*. [21]	386 cases (participants with lower respiratory tract infections were more likely to be BCG unvaccinated i.e. higher odds ratio)
Influenza virus type A (H1N1)	Randomized controlled trial (RCT)	Improved immunogenicity of the H1N1 vaccine, manifesting as increased H1N1 antibody production	Leentjens *et al*. [32]	40 participants (randomized into 20 test and 20 control subjects)
Herpes Simplex Virus (HSV)	Case series	Reduced episodes of HSV infection recurrence	Anderson *et al*. [37]	15 cases
	Case series		Hippman *et al*. [38]	109 cases (all participants remained herpes-free for 4–6 months, 21% after 3 years and 10% after 6 years)

## 4. Mechanism behind BCG-induced protection against viral and non-mycobacterial infections

### 4.1. Heterologous lymphocytic immune response

**Figure 1.**
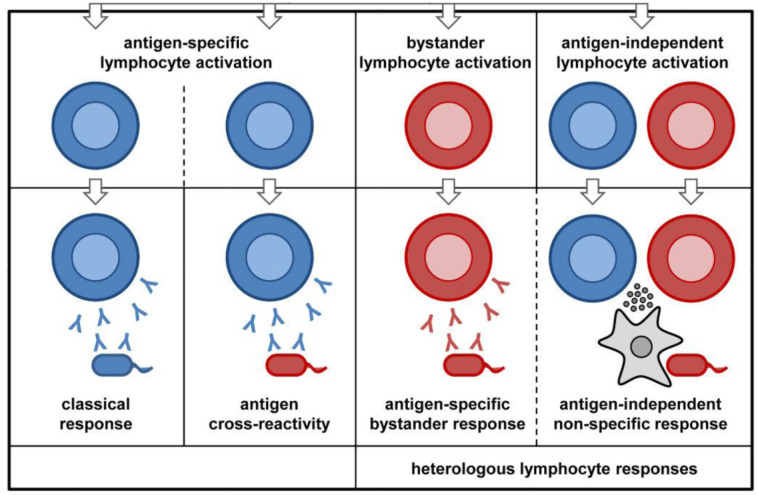
Heterologous lymphocyte responses includes: A. Molecular mimicry between the BCG vaccine and antigens of unrelated pathogens, can cause a cross-reactive immune response to these unrelated antigens (blue coloured antibodies attacking red coloured cells, in the antigen cross-reactivity panel) B. Polyclonal activation of bystander B-/T-cells by BCG vaccination (red coloured antibodies attacking red coloured cells in the bystander lymphocyte activation panel) or C. non-specific lymphocytic response can trigger activation of innate immune cells like macrophages (grey coloured amoeba shaped cells), which secrete cytokines active against unrelated pathogens.

Three main adaptive immune system-dependent mechanisms have been linked to the heterologous effects of the BCG vaccine on viral and non-mycobacterial infections; they include: antigen cross-reactivity, bystander activation of unrelated lymphocytes and lymphocyte dependent activation of innate immunity (Figure 1). Antigen cross reactivity results from molecular mimicry, and may be responsible for some of the off-target effects of the BCG vaccine. Such effects have already been documented for other vaccines such as: the AS03-adjuvanted influenza (H1N1) pandemic vaccine which was associated with the development of narcolepsy in some individuals who received the vaccine. This was due to cross-reactivity with the host antigens, as a result of molecular mimicry between a portion of the influenza antigen (nucleoprotein) and a part of the human brain receptor responsible for promoting wakefulness (hypocretin receptor 2) [39]. It is possible that molecular mimicry between a portion of the BCG vaccine and fragments of other viral pathogens could stimulate the memory T cells to become reactive even to antigens they had not been previously exposed to, as seen in adult humans [40]. It is important to note however that antigen cross-reactivity cannot explain all the many off-target effects seen with the BCG vaccine.

‘Bystander’ response involves the activation of unrelated B cells and/or T cells following an infection or vaccine-induced immune response. This response is often triggered by microbial components or inflammatory cytokines, which cause a non-specific polyclonal activation of T lymphocytes and/or antibody production by B lymphocytes. An example of this, is the stimulation of the production of tetanus-specific antibodies, as well as antibodies against measles and *Toxoplasma gondii* following immunization with tetanus toxoid, which occurs through the polyclonal activation of memory B cells, as reported by Bernasconi *et al*. [41]. In a clinical trial, Kleinnijenhuis *et al*. [42] showed that BCG vaccination resulted in non-specific Th1/Th17 responses, persisting for at least one year post-vaccination. Also, in patients with recurrent respiratory papillomatosis, BCG vaccination successfully triggered a Th1/Th17 immune response [43].

The BCG vaccine is also able to induce a heterologous lymphocytic response which in turn stimulates a strong activation of the innate immune system, manifesting as cytokine production against a secondary, unrelated pathogen. The most common cytokine involved is IFN-γ, from the CD4+ cells. Mathurin *et al*.[44] showed that BCG vaccination was able to protect mice against infection with the vaccinia virus, by stimulating increased IFN-γ production from the CD4+ cells. In addition, some studies have shown that peripheral monocytes collected from the blood post-BCG vaccination, demonstrated increased IFN-γ production upon *in vitro* stimulation with mitogens and other unrelated viral antigens [45].

### 4.2. Innate immune memory

**Figure 2.**
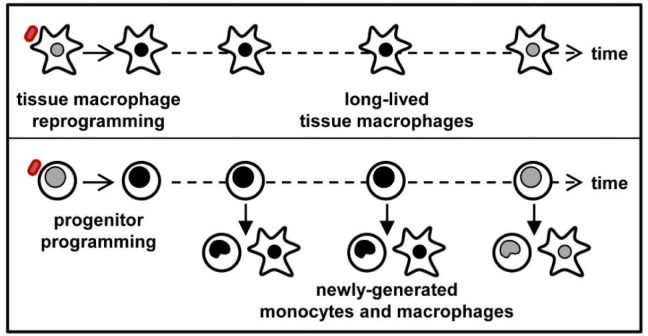
Mechanisms of innate memory in monocytes and macrophages. BCG administration causes epigenetic changes in the innate immune cells (macrophages, monocytes and NK cells), allowing them secrete cytokines–IL-1β and TNF-α in response to unrelated pathogens. This is known as trained immunity or innate immune memory.

It was initially thought that only B- and T-cells could store and initiate memory immune responses; however, we now know that innate immune cells are also able to act as memory cells, through a change in their functional programme following certain infections or vaccinations [46], and the induction of non-specific memory in innate immune cells is known as ‘trained immunity’. This concept is mediated by epigenetic and metabolic changes in the innate immune cells [47]. It has been suggested that the innate immune memory is the main mechanism through which BCG induces most of its non-specific effects, and involves mainly the natural killer (NK) cells, monocytes and macrophages, rather than the T- and B-cells. BCG vaccination enhanced the production of inflammatory cytokines such as: interleukin-1ß (IL-1ß), and tumor necrosis factor (TNF)-α by the peripheral monocytes, for up-to 3 months post-vaccination, upon stimulation by unrelated pathogens *in vitro* [47]. This was accompanied by epigenetic reprogramming of the monocytes at the promoter sites of the genes encoding for these inflammatory cytokines, as well as an increase in production of the activation markers: TLR4, CD14 and CD11b (Figure 2).

Following these findings, Arts *et al*. [36] conducted a randomized controlled trial, to test their hypothesis that the post-BCG vaccination induced rise in cytokine production by monocytes would contribute to better clinical outcomes following an unrelated secondary viral infection. They chose the live attenuated yellow fever vaccine (YFV) as the secondary infecting agent, and randomized the study participants into two groups, the first receiving BCG and the other receiving placebo vaccination. Four weeks later, all subjects received the YFV and the level of viraemia was compared between both groups [36]. They soon observed that subjects in the test groups, who had received the BCG vaccine, had significantly lower levels of the virus than those who received placebo. They also noted that this was associated with epigenetic changes in the circulating monocytes in the test subjects [36]. They then tested for association between trained immunity (measured by the level of induction of production of circulating pro-inflammatory cytokines *ex vivo*) and the levels of yellow fever viraemia, but noted that there was no correlation between post-vaccination increase in IL-6, TNF-α (innate immunity) or IFN-γ, IL-17 (adaptive immunity) and viraemia levels; however, IL-1ß production post-BCG vaccination was a strong predictor of low levels of viraemia in the test patients [36]. This further suggests that the BCG-induced IL-1ß production (through the innate immune memory mechanism) is the main mechanism through which the vaccine exerts its non-specific effects against unrelated viral infections.

The protective role of IL-1ß against viral infections has been confirmed by other independent studies [48]. Both Allen *et al*. [49] and Thomas *et al*. [50] showed that IL-1ß receptor knock-out mouse models, or those lacking the inflammasome components NLRP3, caspase-1 and ASC were highly susceptible to viral infections and had a lower rate of survival following viral challenge.

## 5. Implications of the non-specific effects of BCG on vaccinology

The many non-specific effects of the BCG vaccine could have important implications for vaccination strategies being used currently, against many viral infectious diseases. Due to the evidence that BCG could be an effective prophylactic agent against viral infections, as shown by Arts *et al*., for the yellow fever virus, BCG could be considered for use as prophylaxis against viral infections that cause global pandemics and for which no approved vaccines currently exist. An important example of such diseases is the current SARS-CoV-2 outbreak which has ravaged global public health and the global economy since December 2019 [51]. As we would soon discuss in a subsequent section of this review, certain epidemiological studies have drawn associations between BCG vaccination policies and the morbidity, mortality as well as the transmission patterns of this novel virus, and there have been calls for the BCG vaccine to be considered as a prophylactic measure against infection, particularly for highly susceptible demographics such as: young children, the elderly and frontline health workers [52].

Furthermore, the BCG vaccine could be an important immunotherapeutic agent for the prevention of the reactivation of latent viral infections like: cytomegalovirus (CMV), varicella zoster virus (VZV) and Epstein Barr virus (EBV). Anderson *et al*. [37] reported the successful treatment of recurrent genital herpes with BCG in a small trial of 15 patients, while Hippman *et al*. [38] used the intracutaneous BCG vaccine injection to treat herpes simplex recidivans in a larger study involving 109 patients. All patients remained herpes free for 6 months post-therapy, 21 patients (19%) were herpes free even after 3 years, while 10 patients (9%) were herpes free as at 6 years post therapy [38].

Finally, the ability of the BCG vaccine to induce a strong immunogenic response to other vaccines of unrelated viral pathogens, as described by Arts *et al*. [36], in their study with YFV, and by Scheid *et al*. [53], in their study with Hepatitis B vaccine, opens up the possibility of using the BCG vaccine as an adjuvant, to improve the immunogenicity of vaccines that currently induce suboptimal immune responses, such as the varicella vaccine and the hepatitis B virus (HBV) vaccine. In addition, this mechanism can also be used to develop a new generation of vaccines that make use of BCG to improve their antibody titers and T-cell immune response, such as: the simian immunodeficiency virus vaccine [54], hepatitis C virus (HCV) vaccine [55] and the novel HIV-1 vaccine [56].

## 6. Epidemiological correlation with SARS-CoV-2

In December 2019, an outbreak of a strange cluster of pneumonia was reported from the Huanan Seafood Market, Wuhan, China, which was eventually confirmed to be the coronavirus disease (COVID-19) whose causative agent was the SARS-CoV-2 or the 2019-novel coronavirus [57]. The disease was soon declared a Public Health Emergency of International Concern (PHEIC) by the WHO [58], in January 2020, and subsequently, a full blown pandemic on March 11, 2020. As at August 8, 2020, the disease has spread to 188 countries, causing 19,412,292 infections and 721,925 deaths [59]. Soon after being declared a pandemic, several epidemiological studies emerged, linking the transmission as well as the morbidity and mortality patterns of the virus to the differences in BCG vaccination policies adopted by various countries. This was borne out of the fact that there were certain inexplicable variations in the way and manner the pandemic tore through different parts of the world, as countries with weaker healthcare systems were recording far lower numbers of infections and mortality than projected.

In their study, Miller *et al*. [52] reported that nations with long-standing universal BCG vaccination schemes had recorded significantly lower mortality rates (in deaths per million of the population), as well as transmission rates (in case per million of the population), compared with those who had no such policy in place. They also showed that this effect was strongly influenced by the time of implementation of the policy, with nations like Iran, whose BCG vaccination policy started in 1984, having a higher mortality rate of 19.7 deaths per million, compared with Japan, whose policy started in 1947, having a lower mortality rate of 0.28 deaths per million [52]. Hegarty *et al*. [60] analyzed and matched the case incidence and mortality data from 178 countries with information about their BCG vaccination policies obtained from the BCG world atlas [5], and they reported that those with long-standing BCG vaccination policies had a significantly lower case incidence and mortality rate than those without such policies (38.4 vs. 358.4 cases per million of the population). Similar correlations have been made by Dayal and Gupta [61], Dolgihk [62], Sharma [63], Ebina-Shibuya *et al*. [64] and Kinoshita *et al*. [65]. In a recent analysis, Klinger *et al*. [66] have shown that the protective effect of BCG vaccination is most profound in the younger population (0–24 years), and in those who received the vaccine very recently, while Weng *et al*. [67] showed that COVID-19 patients who had previously been vaccinated with BCG were less likely to require hospitalization, when compared with those who had no previous BCG vaccination, even after adjusting for demographic variations and co-morbidities.

There are however numerous confounding factors that renders this correlation superficial. In a critical review, Kirov highlighted that most of these studies did not accommodate for factors such as: population age, chronic disease states, race, income levels and time from community spread [68], although this is does not apply to the study by Weng *et al*. [67]. He established that median population age had a more significant influence on COVID-19 mortality data than BCG vaccination policy [68]. In addition, according to Hensel *et al*. [69] for countries with higher testing rates of >2500 tests per million of the population, the association between BCG vaccination policy and COVID-19 incidence and mortality data became statistically insignificant. Furthermore, as the pandemic progressed, the virus has gained a stronger foothold in some of the low-resource countries that were referred to in the studies establishing this correlation in the early months of the pandemic, with India and Brazil recording a sharp rise in their case incidence and mortality rates. While some studies have shown that the BCG vaccine has limited effect in protecting against pulmonary TB in adulthood, with efficacy ranging from 0%–80% [8], COVID-19 disproportionately affects the elderly and those with co-morbidities. This population divergence in efficacy casts even more doubts on the possibility that the BCG vaccine would be of significant use in preventing COVID-19 infections, in the most vulnerable, as current arguments suggest. Taking all these factors into considering, it is likely that the proposed efficacy of BCG against COVID-19 might be a case of ecological fallacy rather than scientific fact.

There have been calls for randomized controlled trials investigating the correlation between BCG vaccination and COVID-19 transmission, morbidity and mortality patterns, in order to put a definite scientific conclusion to the argument. Many of such trials are active and ongoing in Australia, the Netherlands and the USA. The Australian study, entitled: ‘BCG Vaccination to Protect Healthcare Workers Against COVID-19 (BRACE)’ (clinical trial number: NCT04327206) [70] is a multicentre, phase III randomized controlled trial involving 10,078 healthcare workers as participants, and aims to investigate the efficacy of the BCG vaccine in reducing the occurrence and severity of COVID-19 infection in healthcare workers. The second trial from the Netherlands, entitled: ‘Reducing Health Care Workers Absenteeism in COVID-19 Pandemic Through BCG Vaccine (BCG-CORONA)’ (clinical trial number: NCT04328441) [71] is a placebo controlled phase III randomized clinical trial involving 1,500 participants and aims to reduce the absenteeism, ICU admissions and death among healthcare workers with direct COVID-19 patient contact. The trial, from the USA, entitled: ‘BCG Vaccine for Health Care Workers as Defense Against COVID-19 (BADAS)’ (clinical trial number: NCT04348370) [72] is a phase IV randomized placebo controlled clinical trial, involving 1800 participants, and aims to test the hypothesis that BCG vaccine can reduce infection and severity and disease in healthcare workers during the epidemic phase of COVID-19. Also, a non-randomized control trial is on-going in India entitled: ‘BCG vaccination in reducing morbidity and mortality in elderly individuals in COVID-19 hotspots’ (clinical trial number: NCT04475302) [73]. It is a phase III trial involving 1450 participants, aged between 60–80 years and reside in designated hotspots for SARS-COV-2 infection in India, including: Mumbai, New Delhi, Chennai and Bhopal. The trial aims to monitor the trend of mortality due to COVID-19 in the elderly following administration of the BCG vaccine. Further data on these trials are provided below:

**Table 2.** Overview of on-going BCG/COVID-19 human trials.

Clinical Trial	Type of study	BCG administration/Doses	Important notes
BRACE Trial (NCT04327206)	Randomized controlled double blinded trial	Adult dose of BCG vaccine, Danish strain 1331, is 0.1 mL injected intradermally (Each 0.1 mL vaccine contains between 200,000 to 800,000 colony forming units). Placebo is 0.1 ml of 0.9% NaCl	Test subjects for this trial could either have previously received the BCG vaccine or not
BCG-CORONA (NCT04328441)	Randomized controlled double blinded trial	Intracutaneous 0.1 mL BCG vaccine, Danish strain 1331, which accounts for 0.075 mg of attenuated Mycobacterium bovis. Placebo is 0.1 mL of 0.9% NaCl solution	Test subjects for this trial could either have previously received the BCG vaccine or not
BADAS Trial (NCT04348370)	Randomized controlled double blinded trial	BCG Tice Strain. Each vial contains 1 × 10^8^ colony forming units. A single dose containing 0.1 ml (2 × 10^5^ CFU) will be administered intradermally to the deltoid. Placebo of 0.1 mL of saline.	Test subjects for this trial could either have previously received the BCG vaccine or not
BCG Vaccine in Reducing Morbidity and Mortality in Elderly Individuals in COVID-19 Hotspots (NCT04475302)	Non-randomized controlled clinical trial	Single-dose BCG vaccine (Freeze-dried). I mLcontains 2 × 10^6^ and 8 × 10^6^ colony forming units with NaCl	Test subjects for this trial could either have previously received the BCG vaccine or not

Sequel to the outcomes of these trials, and the status of ongoing research and production of a COVID-19 vaccine; if the correlation is proven to be true, there would be a need to upscale the production capacity of the BCG vaccine, to ensure its mass availability particularly to the most hard-hit regions, as well as to ensure that supply to countries where tuberculosis is endemic, and which remain heavily on the vaccine to prevent TB infections, is not cut off [74]. In addition, as different countries make use of different strains of the BCG vaccine, research into the efficacy of its effect against COVID-19 has to take into consideration possible differences across strains, to ensure that the most effective strain(s) are distributed across the globe. Notwithstanding, the mode of deployment, in the event of the proven efficacy of the BCG vaccine in controlling the SARS-CoV-2 pandemic, relies largely on its efficiency, and the development of a disease specific vaccine against SARS-CoV-2 should not be halted. However, should the correlation be proven to be non-existent, research into the pathogenicity of SARS-CoV-2 infection, as well as the search for a vaccine, need to be intensified, to help blunt the health impacts as well as economic impacts of the ravaging disease.

## 7. Conclusion

In conclusion, as discussed in this review, there is strong evidence in literature to prove that the BCG vaccine has a lowering effect on all-cause mortality, due to its effect in preventing unrelated viral infections. These effects have been termed non-specific, off-target or heterologous effects, and have been shown to be due in part, to antigen cross-reactivity, bystander activation of polyclonal B- and T-lymphocytes as well as lymphocyte-dependent activation of innate immune response. But perhaps, the most important mechanism behind the several non-specific effects of the BCG vaccine is the activation of innate immune memory, through epigenetic and metabolic reprogramming of circulating monocytes, resulting in an increased production of pro-inflammatory cytokines (particularly IL-1ß) upon stimulation by secondary infections. This has been linked to several implications on future vaccination strategies. It is our opinion that the future of research in this field, lies in understanding further, the mechanism behind these BCG-induced epigenetic changes in innate immune cells, and detailing the exact mechanism of memory response in monocytes and macrophages. Some questions that need to be answered include: how soon these epigenetic changes occur following exposure to the BCG vaccine, and how long do these BCG-induced innate cell memory responses last? Are there differences in the efficacy, onset and duration of BCG-induced innate immune memory responses across the different BCG strains? Outside of what we already know about the immunologic factors that mediate these responses, which other factors are involved and what are their roles in effecting and mediating the epigenetic and metabolic reprogramming of macrophages following BCG vaccination? By answering some of these questions through carefully planned studies, we can further harness trained immunity as a translational immunological principle for the prophylaxis and treatment of the many disease states the BCG vaccine has been touted to have an effect against. In light of the evidence presented above however, it is safe to say that BCG is truly a vaccine with many different faces.
